# Dependence on Nitrogen Availability and Rhizobial Symbiosis of Different Accessions of *Trifolium fragiferum*, a Crop Wild Relative Legume Species, as Related to Physiological Traits

**DOI:** 10.3390/plants11091141

**Published:** 2022-04-22

**Authors:** Astra Jēkabsone, Una Andersone-Ozola, Andis Karlsons, Lāsma Neiceniece, Māris Romanovs, Gederts Ievinsh

**Affiliations:** 1 Department of Plant Physiology, Faculty of Biology, University of Latvia, 1 Jelgavas Str., LV-1004 Rīga, Latvia; astra.jekabsone@lu.lv (A.J.); una.andersone-ozola@lu.lv (U.A.-O.); las.neic8@gmail.com (L.N.); maris.romanovs@lu.lv (M.R.); 2 Institute of Biology, University of Latvia, 4 Ojāra Vācieša Str., LV-1004 Rīga, Latvia; andis.karlsons@lu.lv

**Keywords:** biological nitrogen fixation, biomass partitioning, crop wild relatives, legumes, nitrogen fertilization, photosynthesis-related characteristics, rhizobial symbiosis

## Abstract

Biological nitrogen fixation by legume-rhizobacterial symbiosis in temperate grasslands is an important source of soil nitrogen. The aim of the present study was to characterize the dependence of different accessions of *T. fragiferum,* a rare crop wild relative legume species, from their native rhizobia as well as additional nitrogen fertilization in controlled conditions. Asymbiotically cultivated, mineral-fertilized *T. fragiferum* plants gradually showed signs of nitrogen deficiency, appearing as a decrease in leaf chlorophyll concentration, leaf senescence, and a decrease in growth rate. The addition of nitrogen, and the inoculation with native rhizobia, or both treatments significantly prevented the onset of these symptoms, leading to both increase in plant shoot biomass as well as an increase in tissue concentration of N. The actual degree of each type of response was genotype-specific. Accessions showed a relatively similar degree of dependence on nitrogen (70–95% increase in shoot dry mass) but the increase in shoot dry mass by inoculation with native rhizobia ranged from 27 to 85%. In general, there was no correlation between growth stimulation and an increase in tissue N concentration by the treatments. The addition of N or rhizobial inoculant affected mineral nutrition at the level of both macronutrient and micronutrient concentration in different plant parts. In conclusion, native rhizobial strains associated with geographically isolated accessions of *T. fragiferum* at the northern range of distribution of the species represent a valuable resource for further studies aimed at the identification of salinity-tolerant N_2_-fixing bacteria for the needs of sustainable agriculture, as well as in a view of understanding ecosystem functioning at the level of plant-microorganism interactions.

## 1. Introduction

Nitrogen is a limiting factor for plant growth in many ecosystems, including coastal dunes [1] and salt marshes [2,3]. In the context of coastal habitats, experimental evidence, based on studies in dune grassland models containing legume species, shows that in addition to plant productivity and nitrogen nutrition, legume-rhizobia interaction also determines plant community structure [4]. In turn, biological nitrogen fixation by legume-rhizobacterial symbiosis in temperate grasslands is an important source of soil nitrogen, benefiting non-leguminous species and increasing soil sustainability [5]. For example, the clover species *Trifolium repens* is able to fix 100 to 350 kg N ha^−1^ per year [6]. Due to rising anthropogenic pressure and in the light of global climate changes, research leading to the development of new pasture crops and cultivars having high resilience against unfavorable conditions is of special importance.

Crop wild relative (CWR) species represent an invaluable resource for the increase in genetic diversity of crops aiming at the incorporation of resilience-enhancing adaptations [7,8]. It can be proposed that in the case of legume CWRs, both host plant and symbiotic rhizobacterial diversity needs to be assessed at the functional as well as genetic level because this interaction determines nodulation efficiency and N_2_-fixing ability of a legume-rhizobia symbiosis. However, this important aspect is not usually dissected when analyzing legume CWR resources [9]. Besides, a large practical interest in studies on the legume-rhizobia symbiosis of wild plants native to marginal habitats is related to the need for selecting efficient and durable bacterial strains useful for improving the sustainability of vulnerable agroecosystems [10,11]. 

*Trifolium fragiferum* L. is a CWR legume species, which, being relatively rare in northern Europe, is associated with the European protected habitat ‘Baltic coastal meadow’ [12]. *T. fragiferum* has a number of resilience-related characteristics. Stoloniferous growing habits allow vegetative spread and are associated with the extremely high trampling and cutting tolerance of the species [13]. *T. fragiferum* accessions have considerable tolerance to soil salinity and alkalinity [14,15] as well as can withstand soil waterlogging and flooding [13,16]. In natural conditions, *T. fragiferum* can be found also in soils with relatively low plant-available N concentrations (16–21 mg L^−1^), but the N concentration in plant tissues was at optimum or close to optimum levels, indicating an efficient N_2_-fixing ability of the rhizobial symbiont [17]. Consequently, wild accessions of the species at the northern edge of the distribution range together with their symbiotic bacteria represent a valuable resource for further exploration of the economically valuable characteristics in the context of sustainable agriculture.

In the last decades, emphasis has been given to the characterization of the genetic and functional diversity of a bacterial counterpart of wild legume-rhizobia symbiosis [18,19,20], with significant efforts also in the field of ecosystem functioning [21]. However, plant-related functional aspects of rhizobial symbiosis and symbiotic nitrogen fixation in wild legume species have been only seldom assessed so far. Recently, we showed that rhizobial symbiosis affects adaptation-related physiological processes of coastal dune plant species *Anthyllis maritima* on the background of sand burial and salinity [22]. In addition, rhizobial symbiosis affected the interaction between individuals of *T. fragiferum* and *T. repens*, cocultivated in different substrate salinities, and there was a significant interaction between these factors with respect to plant growth and morphology, including the type of rhizobial bacteria used for inoculation [23].

Photosynthesis-related parameters, such as leaf chlorophyll concentration and chlorophyll-a fluorescence-derived indices of the photochemistry of photosynthesis, have been frequently used to characterize the physiological performance of plants in heterogeneous or unfavorable environmental conditions [24,25,26], including nutrient deficiency [27]. In *Anthyllis maritima*, photosynthesis-related parameters were good indicators of the presence of nodules on plants, as inoculation with native rhizobia led to a significant increase in both leaf chlorophyll concentration as well as fluorescence parameters, such as the Performance Index [22]. In *T. fragiferum,* growth inhibition of asymbiotically cultivated plants was accompanied by a significant decrease in leaf chlorophyll concentration as well as lowered photochemical activity of photosystem II [23].

Legumes can obtain necessary N, either in an inorganic form from soil, or as organic N through symbiotic nitrogen fixation. An actual contribution of each type of N acquisition seems to be both genotype, as well as environment-dependent characteristics [28]. Many studies can be found in the literature comparing the efficiency of rhizobial inoculation vs. nitrogen fertilization on growth and physiological indices of legume crop species [29,30,31,32]. However, there is no comparable information available on the characteristics of functional interactions between different combinations of plant accessions and their native rhizobia in the case of *T. fragiferum*. As a first step to fill this knowledge gap, the aim of the present study was to characterize the dependence of different accessions of *T. fragiferum* from their native rhizobia as well as their response to additional nitrogen fertilization. Changes in growth, biomass partitioning, photosynthesis-related traits and mineral nutrition were used for functional characterization of the outcome of rhizobial symbiosis.

## 2. Materials and Methods

### 2.1. Plants and Microorganisms

Seeds of *T. fragiferum* from eight geographically isolated Latvian micropopulations were used for plant propagation (Table 1). Rhizobia were isolated from nodules collected from roots of two naturally grown plants at the respective micropopulation as described previously [23]. Bacteria were isolated from root nodules of all wild accessions of *T. fragiferum* used in the present study. Seeds of cv. ‘Palestine’ pre-treated with a commercial rhizobial inoculant was obtained from Sheffield’s Seeds Company (Locke, NY, USA) and used for comparison.

### 2.2. Plant Propagation, Cultivation and Treatments

All seed material except part of the seeds of cv. ‘Palestine’ was surface sterilized using a half-diluted commercial bleach (ACE, Procter & Gamble, Warszawa, Poland) for 10 min, followed by three rinses with deionized water (10 min each). Seeds were imbibed in deionized water for 48 h and scarified with a scalpel. Sterilized seeds and part of the seeds of cv. ‘Palestine’ not sterilized were placed in 1 L plastic tissue culture containers on autoclaved garden soil (Biolan, Eura, Finland), closed with lids and cultivated for two weeks in a growth cabinet at 15/20 °C (night/day), photoperiod of 16 h (100 μmol m^−2^ s^−1^ of photosynthetically active radiation). Further transplantation and cultivation in an automated greenhouse were performed as described previously [13]. 

Fully acclimatized four week-old plants were randomly divided into four treatments, five plants per treatment as biological replicates. Asymbiotically cultivated plants were used as controls (C) or were treated with N fertilizer (N) in a form of NH_4_NO_3_ (0.15 g N per container) every even week. Plants inoculated with respective rhizobial suspension were used as symbiotic control (R) or were treated with N fertilizer (RN) in the form of NH_4_NO_3_ (0.15 g N per container) every even week. Each container was inoculated with 6 mL of bacterial suspension (about 10^9^ cells per mL) applying 1 mL of the suspension in six points evenly over the surface of the substrate. Plants from each accession were inoculated with native nodule isolates from wild plants from the same accession. Plants grown from sterilized seeds of cv. ‘Palestine’ were used as an asymbiotic control and for the asymbiotic N-fertilizer treatment, but those grown from non-sterilized seeds were used for rhizobia-inoculated and rhizobia-inoculated + N-fertilized treatments. Every odd week all plants were fertilized with Yara Tera Kristalon Red and Yara Tera Calcinit fertilizers (Yara International, Oslo, Norway). All plants were cultivated in a substrate with 38 mg L^−1^ plant-available N and received additional N from Yara Tera fertilizers (in total, 82.5 mg L^−1^). An individual watering system of containers was used to decrease possible contamination with rhizobial bacteria, with each container having a plastic plate under it for accumulation of excessive water. Additionally, inoculated plants were located on a separate greenhouse table, restricting the chance of physical contact of stolons between individual plants. 

### 2.3. Measurement Measurement of Photosynthesis-Related Parameters

Analysis of photosynthesis-related parameters was started one week after rhizobial inoculation (week 1) and was performed weekly for the next five weeks. For each individual plant, three fully grown photosynthetically active leaves were selected for analysis. Chlorophyll concentration in plant leaves was measured by a chlorophyll meter CCM-300 (Opti-Sciences, Hudson, NH, USA). Results on chlorophyll concentration in week 2 were lost due to technical problems. Chlorophyll *a* fluorescence was measured in leaves dark-adapted for at least 20 min by Handy PEA fluorometer (Hansatech Instruments, King’s Lynn, UK). For the characterization of photochemical activity, the chlorophyll *a* fluorescence parameter Performance Index (total) was used. Performance Index is a complex indicator of photochemical efficiency combining three function-related (trapping of an absorbed exciton, electron transport between the photosystems, reduction of end-electron acceptors) and structure-related (antenna chlorophyll per reaction center chlorophyll) parameters [33]. 

### 2.4. Termination of the Experiment and Measurements

After inoculation with rhizobia, plants were cultivated for seven weeks. Plants were separated into different parts (roots, stolons, leaf petioles, leaf blades, flower stalks, inflorescences). Plant roots were washed individually and the relative degree of nodule presence was evaluated according to the four point scale (0, no nodules; 1, a few nodules (1–5) at only one point; 2, small groups of nodules (<10) at several points on roots; 3, a large number of nodules (>10) throughout the root length). One individual from the asymbiotic control group from each of TF1, TF3, TF4 and TF8 accessions had high presence of nodules and was excluded from further analysis (Table 2). Stolons, leaves and inflorescences were counted, and the length of individual stolons was measured. Plant material was weighed separately before and after drying in an oven at 60 °C for 72 h. 

Mineral element analysis in dry-ashed plant material was performed as described previously [17]. After mineralization of the plant samples and dissolving of the mineral fraction in 3% HCl, chemical analyses were conducted using the following methods: the levels of K, Ca, Mg, Fe, Cu, Zn and Mn were estimated by a microwave plasma atomic emission spectrometer Agilent 4200. Levels of P were analyzed by colorimetry with ammonium molybdate in an acid-reduced medium using a spectrophotometer Jenway 6300. All analyses were performed in triplicate, using representative tissue samples from individual biological replicates. 

### 2.5. Data Analysis

Results were analyzed by KaleidaGraph (v. 5.0, Synergy Software, Reading, PA, USA). Statistical significance of differences was evaluated by one-way ANOVA using posthoc analysis with a minimum significant difference. Principal component analysis, heat map generation and cluster analysis were performed by a freely available web program ClustVis (http://biit.cs.ut.ee/clustvis/; accessed on 19 March 2022) [34]. For principal component analysis, prediction ellipses were such that with a probability of 0.95, a new observation from the same group will fall inside the ellipse. Unit variance scaling was applied to rows; singular value decomposition with imputation was used to calculate principal components. Hierarchical clusters were generated by the average linkage method with correlation distance.

## 3. Results

Root inspection after the experiment revealed that despite precautions taken to sterilize seeds and to prevent bacterial contamination during plant cultivation, several individual asymbiotically grown plants in all *T. fragiferum* accessions except TF6 had nodules on their roots (Table 2). One plant for each of the accessions TF1, TF3, TF45 and TF8 had a large number of nodules throughout the root length. Therefore, these individual plants were excluded from further analysis. No nodules were evident on roots of asymbiotically grown plants receiving N fertilizer except one individual of TF8. All rhizobia-inoculated plants had a relatively high presence of nodules except for one individual of TF1. However, the number of nodules was highly variable for rhizobia-inoculated plants treated with N fertilizer.

Asymbiotically cultivated, mineral-fertilized *T. fragiferum* plants gradually showed signs of nitrogen deficiency, appearing as leaf yellowing, leaf senescence, and a decrease in growth rate. This effect was partially genotype-dependent. The addition of nitrogen, inoculation with native rhizobia, or both treatments significantly prevented the onset of these symptoms. 

**Figure 1:**
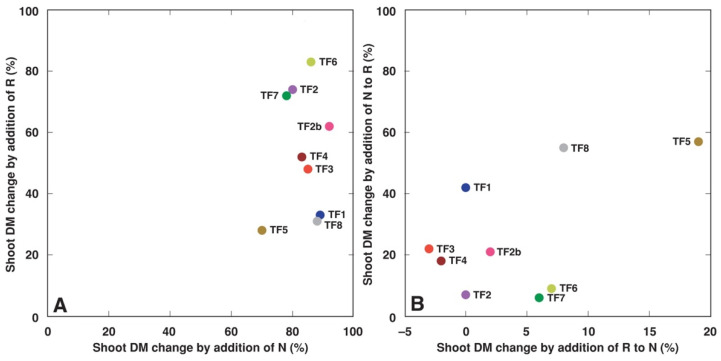
Relative effect of different treatments on shoot dry matter in *Trifolium fragiferum* plants of different accessions. (**A**), effect of separate treatments of N and R; (**B**), effect of N treatment in addition to R and R treatment in addition to N. N, nitrogen; R, rhizobia.

Treatment with additional N fertilizer significantly increased total shoot biomass for all *T. fragiferum* accessions (Table 3). Rhizobial inoculation also had a similar effect for all accessions except TF8. The relative stimulative effect of added N on shoot dry matter accumulation was comparatively similar and ranged from 70% (TF5) to 95% (TF2b) (Figure 1A). However, the stimulative effect of rhizobial inoculation on shoot biomass was rather variable, ranging from 27–35% (TF1, TF5, TF8) to 85% (TF6). Response in biomass changes to rhizobial inoculation of *T. fragiferum* plants receiving N fertilizer was relatively negligible and was not significant for all accessions except TF5 (Table 3), which showed an 18% biomass increase (Figure 1B). Response to N fertilizer of plants inoculated with rhizobia was more variable, ranging from 5 to 57% (Figure 1B), and this effect was significant for TF1, TF2b, TF3, TF4, TF5 and TF8 (Table 3). 

Root growth of *T. fragiferum* plants was relatively less affected by treatments in comparison to shoot growth. Nitrogen treatment resulted in a significant increase of root biomass only for TF2 and TF2b (Table 3). However, rhizobial inoculation or combined treatment did not result in a significant increase in root biomass. 

**Figure 2:**
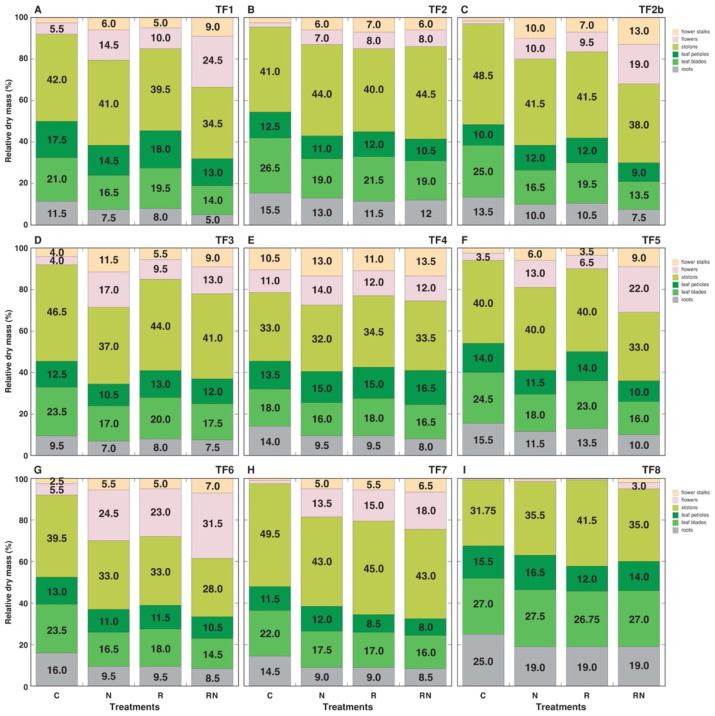
Biomass partitioning in *Trifolium fragiferum* plants of different accessions in control (C), N-fertilized (N), rhizobia-inoculated (R) and rhizobia inoculated + N-fertilized (RN) treatments.

Besides the increase in shoot biomass, another characteristic response of *T. fragiferum* plants to N fertilizer and rhizobial inoculation were changes in biomass partitioning (Figure 2). There were significant differences with respect to the inflorescence number between the accessions already for control plants, ranging from only three in TF7 and TF8 to 26 in TF4 (Table 4). The number of inflorescences significantly increased for all accessions by N fertilizer treatment except TF8, which showed extreme variability between the individual plants. The same effect was evident also for rhizobial inoculation, but it was not statistically significant for TF3 and TF5. For several accessions (TF5, TF6) rhizobial inoculation + N resulted in a higher number of inflorescences in comparison to N fertilization alone. Accordingly, there was an increase in the proportion of biomass in flower stalks and inflorescences by the treatments, which was less pronounced for TF4, which already has high biomass of generative organs in control conditions, and for TF8 with the smallest biomass in generative organs (Figure 2). An increase in partitioning in generative parts was associated with a decrease in biomass proportion in all vegetative parts. However, the tendency to increase the number of leaves was a characteristic response to the treatments, but this effect was not statistically significant for TF2b, TF6 and TF8, due to high variability between individual plants (Table 4). The number of stolons and total stolon length also increased, and the effect was especially pronounced for the plants receiving N fertilizer (Table 5). 

**Figure 3:**
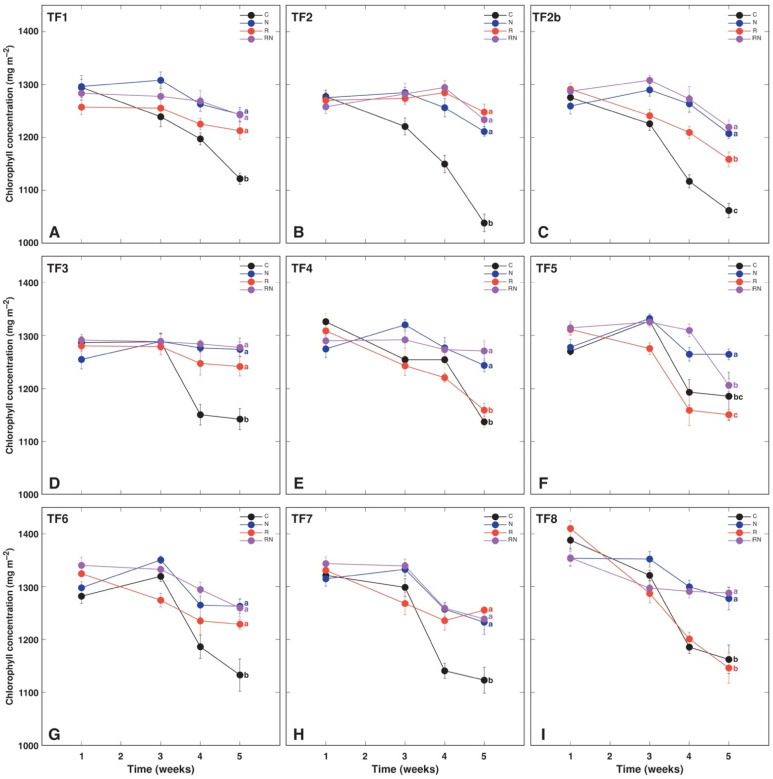
Time course of leaf chlorophyll concentration in *Trifolium fragiferum* plants of different accessions in control (C), N-fertilized (N), rhizobia-inoculated (R) and rhizobia inoculated + N-fertilized (RN) treatments. (**A**) TF1; (**B**) TF2; (**C**) TF2b; (**D**) TF3; (**E**) TF4; (**F**) TF5; (**G**) TF6; (**H**) TF7; (**I**) TF8. Each data point represents mean from 15 independent measurements from five plants ± SE. Different letters of respective color for week 5 results indicate statistically significant differences. Results on week 2 were lost due to technical problems.

**Figure 4:**
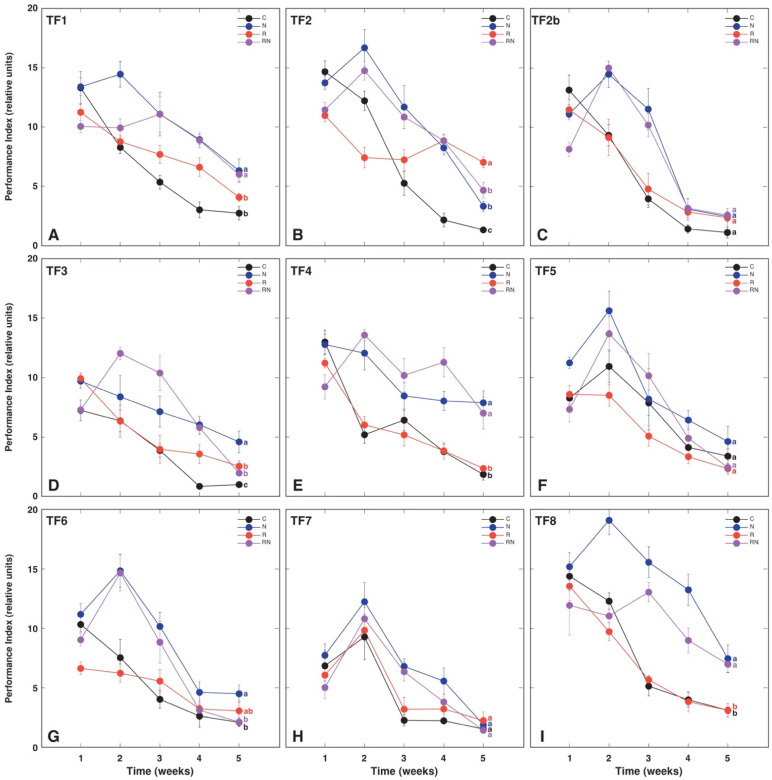
Time course of chlorophyll-a fluorescence parameter Performance Index in *Trifolium fragiferum* plants of different accessions in control (C), N-fertilized (N), rhizobia-inoculated (R) and rhizobia inoculated + N-fertilized (RN) treatments. (**A**) TF1; (**B**) TF2; (**C**) TF2b; (**D**) TF3; (**E**) TF4; (**F**) TF5; (**G**) TF6; (**H**) TF7; (**I**) TF8. Each data point represents mean from 15 independent measurements from five plants ± SE. Different letters of respective color for week 5 results indicate statistically significant differences.

A decrease in leaf chlorophyll concentration with time was a characteristic feature of asymbiotic *T. fragiferum* plants of all accessions (Figure 3). In general, performed treatments prevented this decrease, but the effect varied between accessions and particular treatments. Thus, rhizobial inoculation alone did not prevent a decrease in chlorophyll concentration for TF4 (Figure 3E), TF5 (Figure 3F), and TF8 (Figure 3I). Additionally, for TF2b, a decrease in chlorophyll concentration was more pronounced in rhizobia-inoculated plants in comparison to N-treated plants (Figure 3C). The chlorophyll-a fluorescence parameter Performance Index showed lower resolution ability for different treatments in comparison to that for leaf chlorophyll concentration (Figure 4). In general, the Performance Index tended to be higher for N-fertilized plants, especially, at an early stage of cultivation (week 2), but these differences diminished at the later stages. Only TF2 and TF3 rhizobia-inoculated plants had higher Performance Index values on week 5 in comparison to asymbiotic plants, but N-fertilized plants of several accessions (TF1, TF2, TF3, TF4, TF8) had higher Performance Index values in comparison to control plants at that time. 

**Figure 5:**
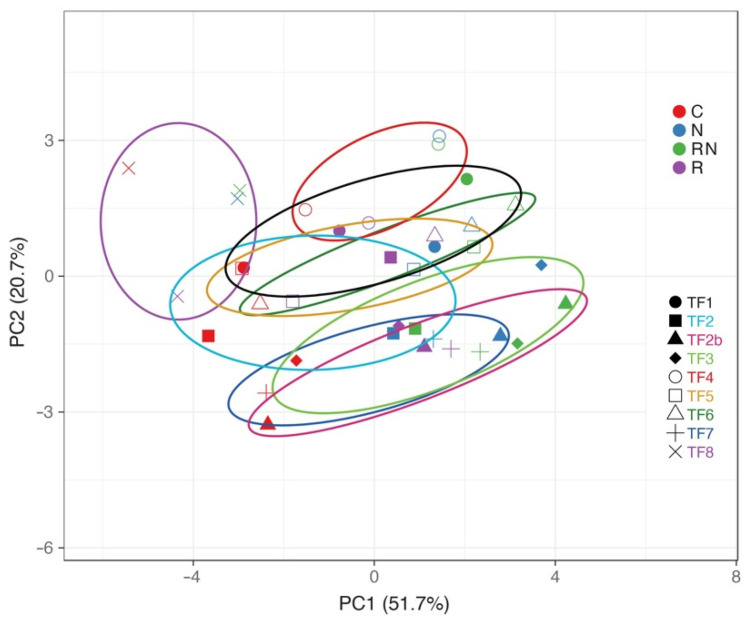
Principal component analysis on the effect of different treatments on morphology, biomass partitioning and physiological indices (chlorophyll concentration and Performance Index on week 5) of *Trifolium fragiferum* plants of different accessions. C, control; N, N-fertilized; R, rhizobia-inoculated; RN, rhizobia inoculated + N-fertilized plants. Prediction ellipses are such that with a probability of 0.95, a new observation from the same group will fall inside the ellipse. Unit variance scaling was applied to rows; singular value decomposition with imputation was used to calculate principal components. X and Y axes show principal component 1 and principal component 2 which explain 51.7% and 20.7% of the total variance, respectively.

**Figure 6:**
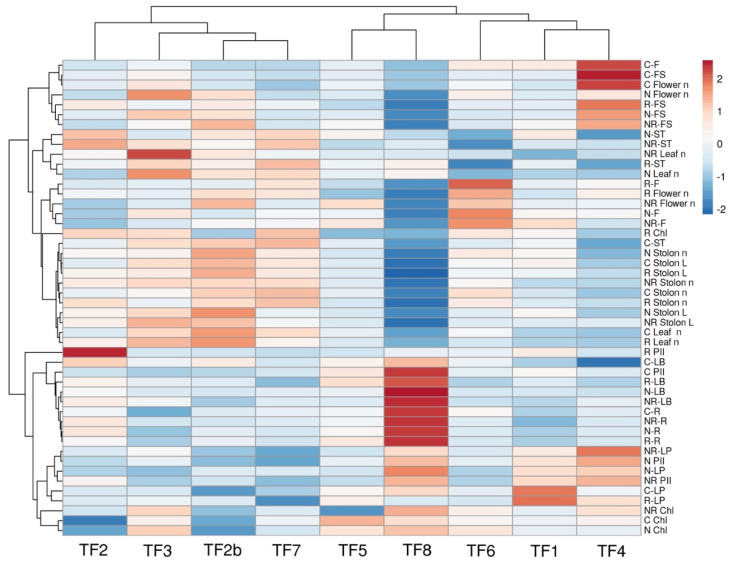
Generated heat map and cluster analysis on effect of different treatments on morphology, biomass partitioning and physiological indices (chlorophyll concentration and Performance Index on week 5) of *Trifolium fragiferum* plants of different accessions. Hierarchical clusters were generated by average linkage method with correlation distance. Color scale shows relative intensity of normalized parameter values. Chl, chlorophyll; F, inflorescences; FS, flower stalks; L, length; LB, leaf blades; LP, leaf petioles; n, number; PI, Performance Index; R, roots; ST, stolons.

**Figure 7:**
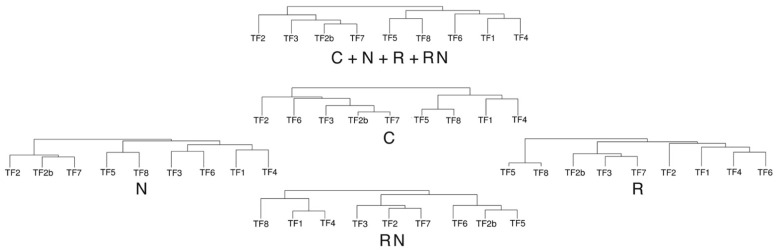
Comparison of results of cluster analysis on morphology, biomass partitioning and physiological indices (chlorophyll concentration and Performance Index on week 5) computed using data from all treatments (C + N + R + RN, the same data set as in Figure 6) or only data from asymbiotic control plants (C), asymbiotic N-fertilized plants (N), rhizobia-inoculated plants (R) or rhizobia-inoculated N-fertilized plants (RN). Hierarchical clusters were generated by average linkage method with correlation distance.

Multivariate analysis was performed to find similarities between accessions in terms of morphological and physiological responses to rhizobial inoculation and N fertilization. It is evident from the results of the principal component analysis that similar responses were characteristic for accessions TF2b, TF3 and TF7; as well as for accessions TF1, TF4, TF5 and TF6 (Figure 5). TF2 had an intermediate position between the two groups, but TF8 showed a unique position. Hierarchical cluster analysis confirmed the similarity of responses between TF2b, TF3 and TF7; as well as between TF1 and TF4; and TF5 and TF8 (Figure 6). It is evident that the tightest association between TF2b and TF7 was related to the close similarity of control plants and, to a lesser extent, N-fertilized and rhizobia-treated plants (Figure 7). The further similarity of TF3 to the previous group was at the level of both control plants and rhizobia-inoculated plants. Association between TF1 and TF4 was justified by a close similarity of rhizobia-inoculated N-treated plants as well as similarity of control and N-treated plants. The connection between TF5 and TF8 predominantly was at the level of both rhizobia-inoculated and control plants. 

**Figure 8:**
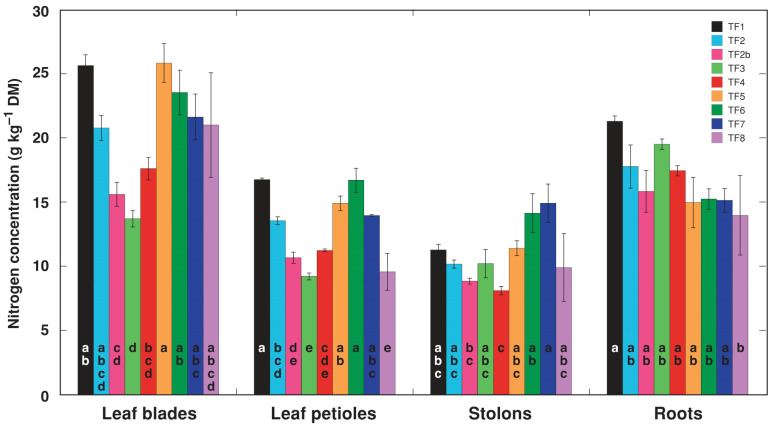
N concentration in various parts of control *Trifolium fragiferum* plants from different accessions. Different letters for a particular plant part indicate statistically significant differences for each plant part.

Nitrogen concentration in different parts of *T. fragiferum* plants grown in asymbiotic conditions was relatively low, and some accession-specific differences were evident (Figure 8). Thus, TF6 had the lowest N concentration among all accessions both in leaf blades and in leaf petioles, while TF1 had the highest concentration in all parts. In general, all treatment types led to an increase in tissue concentration of N, but to various degrees for different accessions (Table 6). The increase was not statistically significant for leaf blades and petioles of TF5 as well as for several other accessions for some treatments. While there was a tendency for increased N concentration in N-treated plants of TF8, the effect was not statistically significant due to extremely high individual variability with respect to this parameter. Moreover, N concentration in symbiotic TF8 plants tended to be lower than in control plants. 

**Figure 9:**
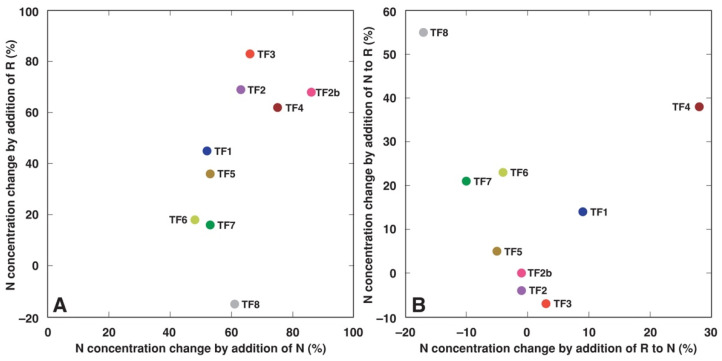
The relative effects of different treatments on average N concentration in *Trifolium fragiferum* plants of different accessions. (**A**) effect of separate treatments of N and R; (**B**) effect of N treatment in addition to R and R treatment in addition to N. N, nitrogen; R, rhizobia.

When the response of average N concentration in plants of different accessions to N fertilizer and rhizobial inoculation was compared, it appeared that the response to N treatment ranged from 48% in TF6 to 86% in TF2b (Figure 9A). The response to rhizobial inoculation ranged from −15% in TF8 to 83% in TF3. Inoculation of N-fertilized plants was relatively ineffective in increasing plant N concentration, with a 28% increase only in TF4 (Figure 9B). Besides, N-fertilization of rhizobia-inoculated plants led to a 38% and 55% increase in average N concentration in TF4 and TF8, respectively.

**Figure 10:**
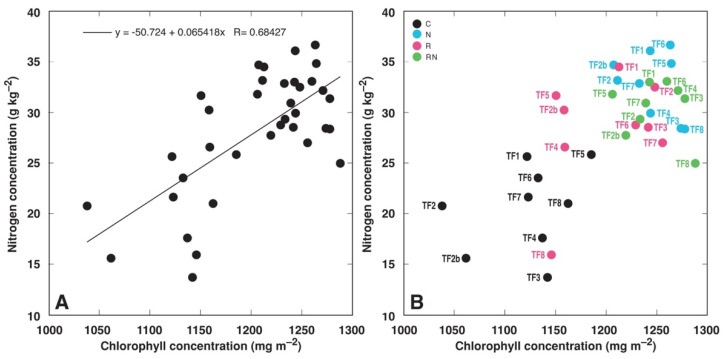
Correlation between leaf blade nitrogen concentration and leaf chlorophyll concentration at day 5. Results are means from three measurements for N analysis and 15 measurements for chlorophyll-analysis. (**A**), unsorted data; (**B**), data sorted by treatments and accessions.

N concentration in leaf blades in general had a moderately tight correlation with leaf chlorophyll concentration (Figure 10A). However, when results were grouped according to different treatments, it appeared that only a low positive correlation was evident for the control group (*R* = 0.308) and rhizobia-inoculated group (*R* = 0.387), but correlation for the N-fertilized group, as well as for rhizobia-inoculated N-fertilized group was negative (*R* = −0.377 and *R* = −0.173, respectively) (Figure 10B).

**Figure 11:**
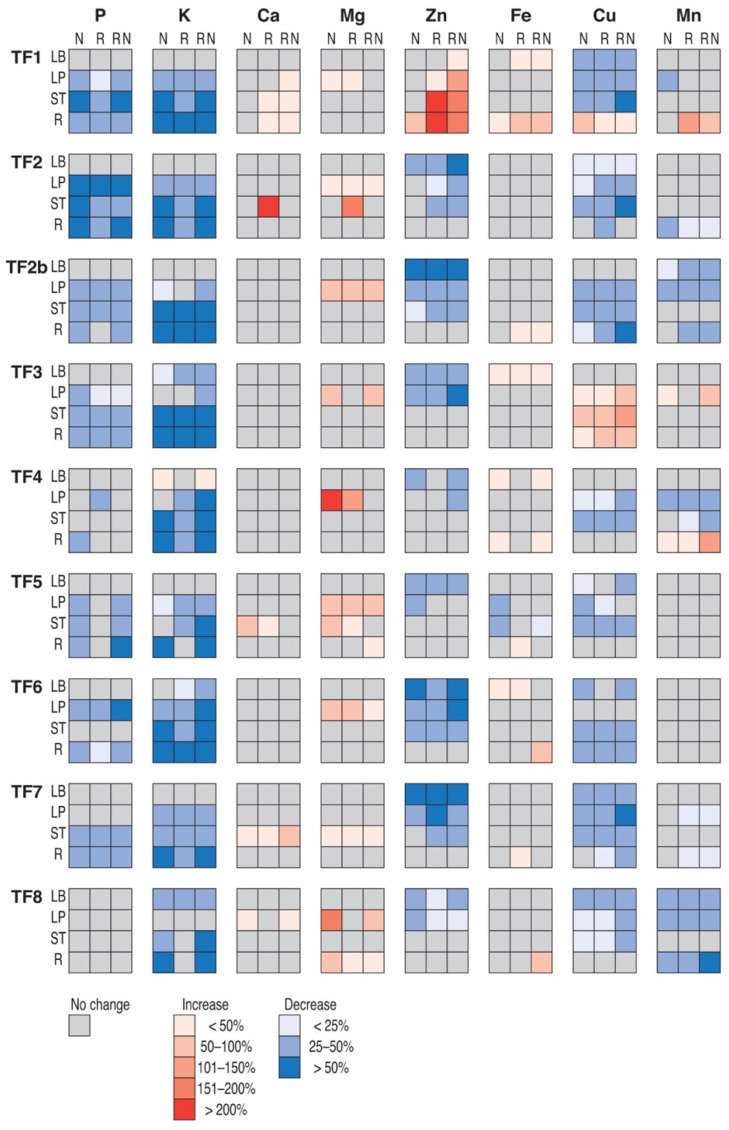
Relative effect of N treatment of asymbiotic plants (N), rhizobial inoculation (R), and N treatment of rhizobia-inoculated plants (RN) on mineral element concentrations in leaf blades (LB), leaf petioles (LP), stolons (ST) and roots (R) of *Trifolium fragiferum* plants of different accessions in comparison to asymbiotic control plants. Only statistically significant effects are taken into the account.

Mineral nutrient concentrations for different parts of control plants are presented in Table S1. Both genotype- and organ-specific changes in mineral nutrient concentration were evident in *T. fragiferum* plants receiving additional N fertilization or inoculated with rhizobia, or by both treatments (Figure 11). Looking at the changes in particular elements, it was seen that some of them were characterized by a predominant decrease in concentration (as for macronutrients P and K) or by a predominant increase in concentration, although to a lesser extent (as for macronutrients Ca and Mg). The concentration of micronutrients Zn and Fe showed a predominant decrease by treatments in all accessions except one (TF1 in the case of Zn and TF3 in the case of Cu responded by increased concentration). Changes for micronutrient Fe were relatively less pronounced, and mostly an increase in concentration was evident. For micronutrient Mn, the effect was rather controversial. Organ specificity of changes was extremely pronounced for P concentration, as no decrease occurred in leaf blades, and, partially, for K concentration. Similarly, Zn concentration did not decrease in roots. There were also some treatment-specific changes in mineral nutrient concentration. Thus, in several accessions, decreases in P and K were less intense in some parts of rhizobia-inoculated plants in comparison to these receiving N fertilizer, or combined treatment. Genotype-specific changes were evident as differences in intensity of changes in concentration of particular mineral element or even as the nature of the change, as for TF1 in the case of Zn and TF3 in the case of Cu, showing an increasing trend of these nutrients in opposite to the rest of genotypes. Multivariate analysis revealed that diversity in mineral nutrient concentration among different accessions increased with performed treatments, resulting in a rather unique mineral element response profile for each plant genotype-rhizobia combination (data not shown). 

## 4. Discussion

### 4.1. Dependence of Growth and N supply on Nitrogen and Rhizobia

In contrast to the majority of plant species, relying on uptake of inorganic soil N, legumes can obtain necessary N, either in an inorganic form, or as organic N through symbiotic nitrogen fixation. In general, the higher amount of N supplied by rhizobial symbiosis affects resource partitioning, resulting in the stimulated growth of symbiotic plants [35]. For *T. fragiferum,* plant nitrogen addition and symbiotic status significantly, and in a genotype-dependent manner, affected biomass partitioning. Most importantly, generative development was highly stimulated, especially, by combination treatment for TF1, TF2b, TF5 and TF6, but this characteristic was not affected in TF8 (Figure 2). For some genotypes, rhizobia inoculation was less effective to increase plant biomass and/or tissue N concentration in comparison to N fertilizer treatment, suggesting the existence of differences in the N_2_-fixing efficiency of the native rhizobia. 

N_2_-fixation efficiency of the established symbiosis can be related to strain genotype [36]. Strain selection (specificity of nodulation) in field conditions can occur at multiple phases of interaction and could be related to infection efficiency at the level of bacterial recognition and nodule development (specificity of nodulation) due to differences in rhizobial competitiveness [37,38,39]. It is important to note, that the level of symbiotic compatibility between clover genotypes and different strains of rhizobia depend on the involvement of multiple genes in both symbiotic partners [40].

In the present study, the two types of treatment, rhizobial inoculation and inorganic nitrogen fertilization led to both increase in plant shoot biomass as well as an increase in tissue concentration of N. The actual degree of each type of response was genotype-specific, but in general, there was no correlation between growth stimulation and increase in tissue N concentration by the treatments. Only TF2 showed a high increase of both shoot biomass and N concentration by rhizobial inoculation. Accessions TF6 and TF7, both with a characteristic low increase of rhizobia-dependent N accumulation (Figure 9A), had a high increase in shoot biomass as a result of rhizobial inoculation (Figure 1A). It seems that these differences in biomass increase vs. N concentration between various accessions of *T. fragiferum* are related to variation in nodule efficiency leading to differences in N metabolism. It is logical to assume that plants from accessions, which growth was most stimulated by rhizobia (TF2, TF6, TF7), had higher demand for N, leading also to a higher total amount of N in plant tissues. It seems that N supply from nodules in TF6 and TF7 was relatively less intense than in TF2, resulting in comparatively lower tissue N concentration. In *T. repens*, additional N fertilization of symbiotic plants resulted in increased biomass accumulation, but total N concentration did not change [41]. In contrast, nodulated *Cicer arietinum* plants that had the largest increase in shoot N, showed an increase in root growth [42]. 

As *T. fragiferum* plants from all these accessions showed better physiological status due to rhizobial inoculation, as evidenced by leaf chlorophyll concentration, it seems that TF2 plants accumulated surplus N in a form of storage compounds, such as nitrate and ammonia in vacuoles or proteins in chloroplasts [43]. N remobilization from storage pools is an important feature for perennial plant survival and resilience [44]. Due to the high proportion of biomass allocated in stolons (Figure 2) and the high potential of rhizobia-dependent N accumulation (Table 6), stolons seem to be a major storage site for N in *T. fragiferum* plants. 

Were there any morphological and physiological differences evident depending on the type of N supply, biological N_2_ fixation vs. inorganic N? Some responses indeed showed treatment-specific characteristics, such as those in total shoot biomass (Figure 1), leaf chlorophyll concentration (Figure 3), and biomass partitioning (Figure 2), of mineral nutrient concentration (Figure 11). From a quantitative point of view, at least for some *T. fragiferum* genotypes, rhizobial symbiosis appeared to be less efficient in comparison to N treatment. In a similar study, rhizobia-inoculated plants of *Trifolium pratense* produced the same biomass as plants receiving additional N fertilizer in a form of ammonium nitrate [45]. It is still not entirely clear what are additional physiological effects of active rhizobial symbiosis on host plants besides N acquisition, but it is established that, at the genetic level, genes responsible for the control of root nodulation intensity, also are involved in the control of root architecture in response to nitrogen [46]. 

It is well-known that a high N supply reduces root nodule formation and/or nitrogen fixation efficiency through the reduction of nitrogenase activity in legumes [11,41,47]. Negative effects of nitrogen have been observed in several stages of symbiosis [48]. In field conditions, additional N fertilizer to dairy pastures had negative effects on the morphology of *T. repens* plants and significantly decreased N_2_-fixation activity [49]. In addition, even symbiotic legume plants favor uptake of mineral N from the soil, as it is an energetically less demanding process [50]. It has been suggested that the lower biomass of N2-fixing plants as compared to N-fertilized plants is attributed to larger respiratory costs in comparison to nitrate assimilation [41]. In contrast, the growth and N uptake rate of *T. repens* cultivars were independent of the availability of mineral N, except at very low N availability levels, with a < 10% biomass reduction [51]. 

### 4.2. Physiological Changes

Leaf chlorophyll content has been already used for the prediction of nodulation efficiency in soybean [52]. It was shown previously that both leaf chlorophyll concentration, as well as chlorophyll-a fluorescence parameter Performance Index, were reliable physiological indicators of rhizobial inoculation in *T. fragiferum*, *T. repens* [23] and *Anthyllis maritima* [22]. In the present study, chlorophyll concentration seemed to be an especially good indicator of physiological changes in *T. fragiferum* plants under the effect of rhizobial inoculation and N fertilizer (Figure 3) in comparison to the Performance Index (Figure 4). However, *T. fragiferum* accessions TF1, TF5 and TF8 showed the least increase in shoot biomass by rhizobial inoculation (Figure 1A). Both TF5 and TF8 indeed did not have differences in leaf chlorophyll concentration between asymbiotic and rhizobia-inoculated plants (Figure 3F,I), but such differences were seen for TF1 (Figure 3A). Moreover, TF4 responded to rhizobial inoculation by a 50% increase in biomass (Figure 1A) but did not show differences in chlorophyll concentration between control and inoculated plants (Figure 3E). 

It might be suggested that changes in leaf chlorophyll concentration do not reflect the degree of growth stimulation but rather changes in leaf N concentration, as has been proposed for many plant species [53,54,55], including legumes [56]. However, the degree of correlation between N and chlorophyll concentration was only moderate (Figure 10), and several accessions with only a low increase in tissue N concentration by rhizobial inoculation, as TF6 and TF7 (Figure 9A), showed significant differences in leaf chlorophyll concentration between control and rhizobia-inoculated plants (Figure 3G,H). It appears that chlorophyll concentration can be used to predict N concentration only in the case of single genotypes or for groups of little diverse genotypes, as has been proposed earlier [57]. 

It is a widely accepted opinion that inoculation with efficient rhizobia, in general, increases the uptake of other mineral nutrients besides N in legume species, as indicated by a rise in the total amount of a particular nutrient on the plant basis [58]. In general, it reflects an increase in demand for mineral elements under conditions of growth intensification. However, when the nutrient concentration in plant tissues is considered, rhizobial inoculation may cause different changes. It needs to be emphasized that apart from the physiological effects of symbiosis on nutrient uptake, rhizobia can affect nutrient availability to plants in the rhizosphere through P and Fe solubilization by acidification as well as siderophore release for Fe^3+^ absorption [35]. 

In the leaves of *Trifolium pratense*, the concentration of Mg, Fe, Mn, and Cu increased, but that of P decreased [59]. In the leaves of *Vigna unguiculata*, the concentration of all macro and micronutrients increased, but the degree of increase was bacterial strain-dependent [60]. In the leaves of *Vicia faba*, the concentration of Mg, P, K, Ca, Zn increased; and in leaves of *Glycine max*, the concentration of Mg, K, Ca, Fe, Cu, Zn increased, but that of Mn decreased [61]. The decrease in macronutrient P and K concentration in *T. fragiferum* plants noted in the present study was largely organ-specific, as for a majority of accessions, the concentration of these elements in leaf blades remained unaffected by rhizobial inoculation and N treatment (Figure 11). Consequently, the level of these two essential nutrients was tightly regulated and redistributed to photosynthetic tissues, and leaf blades, but their decrease in other plant parts reflected the dilution of these nutrients due to an increase in total biomass. Interestingly, these effects were also treatment-specific, as symbiotic plants without additional N fertilization showed a less intense decrease in P and K concentration, at least, for several accessions (Figure 11). 

Variation in mineral nutrient concentrations in tissues of *T. fragiferum* was highly increased by rhizobia inoculation and N treatment. In the cultivated legumes *Vicia faba* and *Glycine max,* inoculation with a commercial preparation of rhizobia resulted in significant changes in the concentration of several mineral elements in plant leaves, but the observed changes were usually only up to 20% [61]. In contrast, in the present study, the increase in the concentration of several nutrients exceeded 200%, and the decrease for several nutrients was more than 50% (Figure 11). These differences could be related to the fact that wild legume species, like *T. fragiferum*, could be more dependent on rhizobial symbiosis in comparison to legume crop species. Expansion of the mineralome in *T. fragiferum* plants was observed also as a result of increasing salinity, leading to the establishment of a genotype-specific mineral element response trend within a plant, and it was suggested to reflect homeostasis maintenance-related adaptive response [15]. 

### 4.3. Ecological Implications

Environmental factors are significant modifiers of legume-rhizobia interactions. Thus, both light intensity and level of mineral nutrient availability are important for the regulation of biomass accumulation in symbiotic *Glycine max* plants [62]. When the light was a limiting factor, rhizobial symbiosis did not affect above-ground biomass and even decreased root biomass. In addition, rhizobia increased plant biomass only in low nutrient conditions. However, in this type of study, the total level of mineral nutrients was manipulated instead of adding surplus nitrogen on the background of optimum (or low) mineral availability level. The critical importance of light in the modulation of rhizobial symbiosis has been associated with the fact that light-dependent photosynthesis-driven carbon acquisition represents a resource invested in nodule development further leading to a higher N supply from symbiosis [28]. 

It is generally believed that the growth of legumes in saline conditions is more sensitive than rhizobial growth, but usually, nodulation, as well as N2-fixation activity, is reduced at near 0.3% salinity [63]. It is possible that increased salinity also indirectly negatively affects symbiotic nitrogen fixation, associated with decreased transport rate of photosynthates from the host plant to nodules as a result of inhibition of photosynthesis and/or growth by salinity. However, usually a negative response of photosynthesis to salinity is less pronounced than that of nitrogen fixation [64].

In natural conditions under high salinity, which is characteristic also for *T. fragiferum* habitats in Northern Europe, effective salt-tolerant nodules are expected to be found, reflecting the role of rhizobial symbiosis in adaptation to particular environmental conditions [65]. Effective salt-tolerant bacterial strains isolated from *T. fragiferum* need to be further explored for their potential to improve agricultural sustainability in saline soils. It has been already shown that the presence of rhizobial symbiosis modulates the interaction between *T. fragiferum* and *T. repens* on the background of increased soil salinity [23]. Moreover, in natural conditions, legume plants are involved in tripartite interaction with both rhizobial bacteria, as well as arbuscular mycorrhizal fungi, with mycorrhizal symbiosis having strong effects on nodulation [66]. It seems to be important that the degree of mycorrhizal symbiosis in *T. fagiferum* plants is affected by changes in soil salinity [67]. 

Biomass allocation is a characteristic plastic response of both stoloniferous and rhizomatous clonal plants to changing resource availability [68]. For stoloniferous plants, an increase in nutrient availability usually results in increased clonal growth without a decrease in sexual reproduction, which is in striking contrast with the results of the present study, showing that allocation to generative structures is a typical response to increased N availability. However, genotype-dependent variation in this trait was evident, even among accessions with high growth stimulation by N fertilization and rhizobial inoculation, as accession TF6 had the highest contribution of generative organs in increasing total biomass by N treatment + rhizobia inoculation (31.5%), but TF2 reached only 8.0% (Figure 2). 

### 4.4. Limitations of the System and Future Perspectives

In this type of experiment, it is important that control plants do not develop rhizobial symbiosis for as long as possible. How efficient was the exclusion of rhizobial symbiosis in control, “asymbiotic” plants? For all accessions except TF6, at least one individual of control plants had symbiotic nodules on roots, but there were four plants of cv. ‘Palestine’ (TF8) having small groups of nodules at several points or even a large number of nodules throughout the root length (Table 2). It is most likely that the sterilization procedure used was not efficient enough to get rid of all bacterial cells from the seed surface, as this seed material was preinoculated. However, these bacteria seemed to be inefficient in N_2_ fixing, as evidenced by decreasing trend of leaf chlorophyll concentration in both control and inoculated plants of TF8 (Figure 3I), as well as a lack of significant effect of rhizobia on shoot biomass (Table 3). For other accessions, the presence of nodules on roots of non-inoculated plants most likely resulted from accidental bacterial contamination at the late stages of cultivation and most likely had little effect on plant nitrogen supply.

Usually, plant genotype dependence on symbiotic performance is evaluated for plants grown in an N-free medium, allowing to estimate of the amount of fixed N directly from N analysis in shoots [40]. In the present study, for practical reasons, control plants received a near-optimal level of full mineral fertilizer, and it was revealed that the addition of surplus nitrogen could completely replace rhizobial inoculation. On the other hand, strain genotype effects cannot be excluded. Additional studies aimed at revealing of genetic and functional diversity of rhizobial symbionts are necessary in order to fully understand differences found in different *T. fragiferum* accessions with respect to their native bacterial symbionts, as one of the most important factors shaping their characteristic growth responses in highly heterogeneous conditions. The extremely important ecophysiological aspect of the rhizobial symbiosis of *T. fragiferum* is related to interspecies competition in saline habitats, as shown by a previous study [23].

One of the prominent features of wild legume species is the ability to establish a symbiosis with bacterial strains belonging to different taxonomic groups [18]. However, it is usually suggested that clover species have developed a symbiosis with highly specific strains of rhizobia. Thus, a strain isolated from *Trifolium repens* were able to induce root nodule formation on *T. repens*, but not on *Lotus corniculatus* or *Ononis repens* [4]. In contrast, several strains of *Rhizobium leguminosarum, Bradyrhizobium japonicum* and *Mesorhizobium* sp. have been isolated from root nodules of *T. fragiferum* growing in subtropical zones of China [69] pointing to the existence of a large diversity of symbiotic rhizobia associated with the species. Similarly, nodules of *Trifolium pratense* plants growing in a close vicinity contained *Rhizobium* isolates with high genetic diversity (eight genetically distinct groups) and metabolic variability with respect to carbon and energy sources [70]. As rhizobia were isolated from root nodules of a limited number of *T. fragiferum* plants per accession in the present study, the obtained results on differences in the degree of symbiotic dependence may not fully reflect the efficacy of symbiotic interactions in the natural population. 

Particular rhizobial strains could differ both in their competitiveness as well as N_2_-fixing efficiency. Competition for nodule colonization can be tough, with metabolically more versatile strains (being able to use a wider range of energy-providing substrates) usually being more competitive [71]. However, the metabolic versatility of a particular bacterial strain does not at the same time provide the efficiency of symbiosis [70]. For *Trifolium* species, bacterial strains with effective N_2_ fixation have been shown to be also more competitive for nodule occupancy [72]. The same relationship was found also for other legume species [73]. Additionally, the effects of active symbiosis on plant responses to abiotic stress can be dependent on rhizobia genotype [23]. Consequently, further studies need to address the problem of taxonomic and biological relatedness of native bacterial strains found in geographically isolated *T. fragiferum* accessions.

## 5. Conclusions

All tested combinations of crop wild relative *T. fragiferum* accessions vs. their native rhizobial types were relatively specific with respect to plant functional responses to N availability and inoculation. The dependence of biomass accumulation on N fertilization was similarly high in all accessions (70 to 95%), but the dependence of inoculation with native rhizobia was more variable (27 to 85%). Based on the similarity of morphophysiological responses, three groups of plant/rhizobia combinations were evident, but most of the relationships were at the level of control plant and N-treated plant characteristics. Native rhizobial strains associated with geographically isolated accessions of *T. fragiferum* at the northern range of distribution of the species represent a valuable resource for further studies aimed at the identification of stress-tolerant symbiotic N_2_-fixing bacteria for the needs of sustainable agriculture as well as in a view of understanding of ecosystem functioning at the level of plant-microorganism interactions. 

## Figures and Tables

**Table 1:** Geographically isolated micropopulations (accessions) of *Trifolium fragiferum* in Latvia were analyzed in the present study.

Code	Associated Water Reservoir	Habitat	Location	Coordinates
TF1	Lake Liepājas	Salt-affected wet shore meadow	City of Liepāja	56°29′29″ N, 21°1′38″ E
TF2	River Lielupe	Salt-affected shore meadow	City of Jūrmala, Lielupe, River Lielupe Estuary	57°0′11″ N, 23°55′56″ E
TF2b	River Lielupe	Shore meadow	City of Jūrmala, Majori	56°57′29″ N, 23°49′11″ E
TF3	River Buļļupe	Shore meadow	City of Rīga, Kurzeme District, Island of Buļļu Sala, Vakarbuļļi	56°59′53″ N, 23°57′21″ E
TF4	–	Degraded urban land	City of Rīga, Vidzeme Suburb	56°57′46″ N, 24°7′2″ E
TF5	The Gulf of Riga of the Baltic Sea	Salt-affected wet coastal meadow	Salacgrīva Parish, Randu Meadows	57°49′51″ N, 24°20′12″ E
TF6	The Gulf of Riga of the Baltic Sea	Salt-affected wet coastal meadow	Salacgrīva Parish, Randu Meadows	57°50′9″ N, 24°20′15″ E
TF7	The Gulf of Riga of the Baltic Sea	Dry coastal meadow	Town of Ainaži	57°52′8″ N, 24°21′10″ E
TF8 cv. ‘Palestine’	na	na	na	na

na, not available.

**Table 2:** Relative degree of nodule presence on roots of individual plants of *Trifolium fragiferum* by treatments.

	C		N		R		RN	
Accession	By Replicates	Mean ± SE	By Replicates	Mean ± SE	By Replicates	Mean ± SE	By Replicates	Mean ± SE
TF1	2-0-0-0-3	1.0 ± 0.6	0-0-0-0-0	0	3-1-3-3-3	2.6 ± 0.4	0-0-3-0-0	0.6 ± 0.6
TF2	0-2-0-0-1	0.6 ± 0.4	0-0-0-0-0	0	3-3-2-3-3	2.8 ± 0.2	1-1-0-1-0	0.6 ± 0.3
TF2b	0-1-0-0-0	0.2 ± 0.2	0-0-0-0-0	0	3-3-3-3-3	3.0 ± 0.0	3-2-1-1-1	1.6 ± 0.4
TF3	2-0-3-1-0	1.2 ± 0.6	0-0-0-0-0	0	3-3-3-3-3	3.0 ± 0.0	2-1-1-1-2	1.4 ± 0.3
TF4	3-0-0-0-0	0.6 ± 0.6	0-0-0-0-0	0	3-3-3-3-2	2.8 ± 0.2	0-0-0-0-0	0
TF5	0-2-1-0-0	0.6 ± 0.4	0-0-0-0-0	0	2-2-3-3-3	2.6 ± 0.3	0-2-1-0-0	0.6 ± 0.4
TF6	0-0-0-0-0	0	0-0-0-0-0	0	3-2-3-3-2	2.6 ± 0.3	0-0-1-1-2	0.8 ± 0.4
TF7	2-0-0-1-2	1.0 ± 0.5	0-0-0-0-0	0	3-2-3-3-2	2.6 ± 0.3	0-0-0-0-0	0
TF8	2-2-0-3-2	1.8 ± 0.5	0-0-1-0-0	0.2 ± 0.2	3-2-2-3-3	2.6 ± 0.3	0-0-0-0-0	0

0, no nodules; 1, a few nodules at only one point; 2, small groups of nodules at several points on roots; 3, a large number of nodules throughout the root length. C, asymbiotic control; N, asymbiotic N-fertilized; R, rhizobia-inoculated; RN, rhizobia-inoculated N-fertilized plants.

**Table 3:** Total dry mass (DM) of above-ground parts and roots in control (C), N-fertilized (N), rhizobia-inoculated (R) and rhizobia inoculated + N-fertilized (RN) plants of *Trifolium fragiferum*.

	Shoot DM (g Plant^−1^)				Root DM (g Plant^−1^)			
Accession	C	N	R	RN	C	N	R	RN
TF1	11.4 ± 0.3 c	21.6 ± 0.4 a	15.2 ± 0.5 b	21.6 ± 0.7 a	1.48 ± 0.14 a	1.72 ± 0.25 a	1.36 ± 0.13 a	1.21 ± 0.14 a
TF2	10.7 ± 0.7 b	19.9 ± 1.1 a	18.6 ± 0.4 a	20.0 ± 1.6 a	1.96 ± 0.20 b	3.01 ± 0.19 a	2.42 ± 0.16 ab	2.63 ± 0.28 ab
TF2b	12.2 ± 0.3 c	23.4 ± 0.9 a	19.7 ± 0.8 b	23.8 ± 0.4 a	1.93 ± 0.15 b	2.65 ± 0.14 a	2.34 ± 0.16 ab	1.97 ± 0.14 b
TF3	14.8 ± 0.7 c	27.4 ± 0.7 a	21.9 ± 1.1 b	26.7 ± 0.7 a	1.51 ± 0.11 a	2.06 ± 1.12 a	1.84 ± 0.25 a	2.18 ± 0.17 a
TF4	12.8 ± 0.7 c	23.3 ± 0.4 a	19.4 ± 0.9 b	22.9 ± 0.9 a	2.06 ± 0.19 a	2.49 ± 0.12 a	2.07 ± 0.20 a	2.02 ± 0.17 a
TF5	12.0 ± 0.3 d	20.3 ± 1.5 b	15.4 ± 0.6 c	24.1 ± 0.4 a	2.20 ± 0.20 a	2.73 ± 0.26 a	2.27 ± 0.08 a	2.96 ± 0.25 a
TF6	10.0 ± 0.2 b	18.7 ± 0.7 a	18.4 ± 0.3 a	20.0 ± 0.7 a	1.88 ± 0.17 a	1.93 ± 0.07 a	1.96 ± 0.07 a	1.89 ± 0.16 a
TF7	9.9 ± 0.6 b	17.7 ± 0.9 a	17.0 ± 0.6 a	18.0 ± 1.0 a	1.69 ± 0.24 a	1.69 ± 0.20 a	1.69 ± 0.14 a	1.72 ± 0.04 a
TF8	10.2 ± 0.7 b	19.1 ± 1.3 a	13.3 ± 1.7 b	20.6 ± 1.3 a	3.38 ± 0.42 a	4.74 ± 0.12 a	3.12 ± 0.40 a	4.68 ± 0.63 a

Different letters indicate statistically significant differences (*p* < 0.05) between treatments.

**Table 4:** Number of flowers and number of leaves in control (C), N-fertilized (N), rhizobia-inoculated (R) and rhizobia inoculated + N-fertilized (RN) plants of *Trifolium fragiferum*.

	Flowers (*n* Plant^−1^)				Leaves (*n* Plant^−1^)			
Accession	C	N	R	RN	C	N	R	RN
TF1	6. 0 ± 1.2 c	38.4 ± 4.3 ab	22.5 ± 5.1 b	51.8 ± 9.1 a	293 ± 15 b	489 ± 43 a	427 ± 42 ab	390 ± 25 ab
TF2	8.6 ± 1.4 b	32.0 ± 4.6 a	33.0 ± 5.4 a	34.2 ± 7.8 a	339 ± 19 c	489 ± 25 b	590 ± 29 a	587 ± 8 a
TF2b	6.4 ± 1.9 b	63.8 ± 8.4 a	45.4 ± 9.2 a	83.0 ± 14.1 a	531 ± 22 a	612 ± 56 a	756 ± 81 a	635 ± 78 a
TF3	14.5 ± 4.8 c	81.6 ± 6.0 a	30.4 ± 7.6 bc	48.4 ± 9.1 b	470 ± 36 b	687 ± 51 ab	709 ± 71 ab	854 ± 89 a
TF4	26.3 ± 3.3 c	58.6 ± 2.0 a	38.4 ± 2.2 b	54.0 ± 5.3 a	277 ± 19 c	485 ± 12 a	412 ± 22 b	461 ± 11 ab
TF5	10.0 ± 2.7 c	39.0 ± 5.0 b	15.6 ± 2.4 c	74.2 ± 5.5 a	361 ± 16 b	544 ± 27 a	479 ± 30 a	515 ± 31 a
TF6	11.0 ± 1.7 c	54.6 ± 6.5 b	55.8 ± 3.3 b	80.0 ± 6.9 a	384 ± 32 a	465 ± 9 a	472 ± 20 a	469 ± 37 a
TF7	3.0 ± 1.0 b	31.8 ± 3.9 a	42.2 ± 5.0 a	47.8 ± 8.5 a	462 ± 26 b	631 ± 26 a	584 ± 35 a	561 ± 15 ab
TF8	3.0± 3.0 a	10.8± 5.5 a	1.6± 1.6 a	15.8± 7.8 a	233± 18 a	578± 87 a	367± 88 a	501 ± 87 a

Different letters indicate statistically significant differences (*p* < 0.05) between treatments.

**Table 5:** Number of stolons and total stolon length in control (C), N-fertilized (N), rhizobia-inoculated (R) and rhizobia inoculated + N-fertilized (RN) plants of *Trifolium fragiferum*.

	Stolons (*n* Plant^−1^)				Stolon Length (m Plant^−1^)			
Accession	C	N	R	RN	C	N	R	RN
TF1	27.7 ± 1.4 b	57.8 ± 4.1 a	37.8 ± 4.6 b	43.0 ± 3.4 ab	10.7 ± 0.7 c	18.2 ± 0.6 a	13.8 ± 1.2 bc	14.5 ± 0.6 b
TF2	31.4 ± 0.9 b	57.8 ± 2.1 a	52.6 ± 1.8 a	61.2 ± 2.7 a	9.0 ± 0.7 b	18.9 ± 1.4 a	15.0 ± 0.8 a	18.1 ± 0.9 a
TF2b	42.2 ± 4.4 c	73.5 ± 3.1 a	53.0 ± 3.1 bc	67.8 ± 5.4 ab	13.9 ± 0.9 c	24.9 ± 1.1 a	18.8 ± 0.8 b	22.0 ± 1.3 ab
TF3	34.0 ± 2.1 c	59.0 ± 3.4 ab	41.4 ± 3.8 bc	67.8 ± 7.4 a	12.5 ± 0.2 c	21.6 ± 2.3 ab	15.7 ± 1.1 bc	22.7 ± 1.5 a
TF4	23.2 ± 2.6 c	41.0 ± 1.3 a	34.2 ± 1.6 b	42.6 ± 0.9 a	6.7 ± 0.3 c	13.1 ± 0.4 a	11.2 ± 0.4 b	14.5 ± 0.5 a
TF5	30.6 ± 1.2 b	49.4 ± 3.6 a	36.2 ± 2.3 b	49.4 ± 3.2 a	8.8 ± 0.6 b	15.4 ± 2.2 a	12.7 ± 1.0 ab	14.2 ± 1.4 ab
TF6	40.0 ± 2.9 b	61.0 ± 5.0 a	48.4 ± 2.0 ab	56.8 ± 4.9 a	10.3 ± 0.3 b	16.1 ± 0.6 a	14.0 ± 0.3 a	14.5 ± 0.9 a
TF7	43.6 ± 1.8 c	61.8 ± 1.4 ab	56.8 ± 2.3 b	66.4 ± 1.8 a	11.9 ± 0.1 b	16.8 ± 0.5 a	16.0 ± 0.8 a	16.5 ± 1.0 a
TF8	15.3± 1.8 b	32.8± 4.1 a	18.0± 3.1 b	25.8± 1.7 ab	3.3± 0.7 b	9.3± 1.4 a	5.8± 1.1 ab	6.2± 1.3 ab

Different letters indicate statistically significant differences (*p* < 0.05) between treatments.

**Table 6:** Changes in concentration of nitrogen (% in comparison to asymbiotic control) in various parts of different accessions of *Trifolium fragiferum* plants, treated asymbiotically with nitrogen(N), incolutaed with rhizobia (R), inoculated with rhizobia and treated with nitrogen (RN).

	Leaf Blades			Leaf Petioles			Stolons			Roots		
Accession	N	R	RN	N	R	RN	N	R	RN	N	R	RN
TF1	141 *	135 *	129 *	127 *	128 *	131 *	207 *	170 *	257 *	131	148 *	155 *
TF2	152 *	156 *	141 *	122 *	130	143 *	221 *	228 *	222 *	158 *	163 *	141 *
TF2b	222 *	194 *	178 *	181 *	156 *	148 *	167 *	153 *	224 *	174 *	170 *	170 *
TF3	208 *	208 *	229 *	173 *	182 *	178 *	151 *	181 *	147 *	130 *	161 *	131 *
TF4	170 *	151 *	183 *	147 *	116	153 *	252 *	237 *	485 *	132	144 *	139 *
TF5	135	123	123	113	111	120	211 *	156 *	163 *	153 *	152 *	156 *
TF6	156 *	122	141 *	106	98	102	156 *	102	168 *	175 *	148 *	158 *
TF7	152 *	125	143 *	142 *	101	118	150 *	92	123	167 *	144 *	169 *
TF8	135	76	119	156	96	158	220	100	134	134	67	109

Significant changes from the respective control values are indicated by *.

## Data Availability

All data reported here is available from the authors upon request.

## Supplementary Materials

The following supporting information can be downloaded at: https://www.mdpi.com/article/10.3390/plants11091141/s1, Table S1: Mineral nutrient concentration in different parts of control *Trifolium fragiferum* plants from different accessions.
