# Supplementary material for: Dependence on Nitrogen Availability and Rhizobial Symbiosis of Different Accessions of Trifolium fragiferum, a Crop Wild Relative Legume Species, as Related to Physiological Traits
Source: Plants (Basel). 2022 Apr 22;11(9):1141. doi: 10.3390/plants11091141 (PMC9099520; doi:10.3390/plants11091141)
Supplement: Supplementary file 1 [file plants-11-01141-s001.zip › plants-1674526-supplementary.pdf]

Table S1. Mineral nutrient concentration in different parts of control *Trifolium fragiferum* plants from different accessions.

Nutrient (unit)	Plant part	TF1	TF2	TF2b	TF3	TF4	TF5	TF6	TF7	TF8
P (g kg^{-1} DM)	Leaf blades	2.57 ± 0.15	2.20 ± 0.06	2.93 ± 0.07	2.13 ± 0.03	2.03 ± 0.20	2.90 ± 0.10	2.91 ± 0.17	2.87 ± 0.02	2.87 ± 0.33
	Leaf petioles	2.85 ± 0.03	3.55 ± 0.09	3.62 ± 0.24	2.03 ± 0.09	2.00 ± 0.06	2.77 ± 0.07	3.47 ± 0.37	2.45 ± 0.19	2.07 ± 0.03
	Stolons	4.03 ± 0.55	3.13 ± 0.29	3.16 ± 0.08	2.93 ± 0.12	2.37 ± 0.15	3.57 ± 0.32	1.78 ± 0.76	3.62 ± 0.04	2.63 ± 0.13
	Roots	2.85 ± 0.03	3.50 ± 0.10	3.03 ± 0.15	3.00 ± 0.46	2.50 ± 0.10	3.60 ± 0.06	3.36 ± 0.05	3.63 ± 0.10	2.65 ± 0.13
K (g kg^{-1} DM)	Leaf blades	11.53 ± 0.86	10.37 ± 0.73	10.63 ± 0.56	15.53 ± 6.02	8.83 ± 0.58	10.83 ± 0.48	12.13 ± 0.29	9.77 ± 0.37	14.87 ± 5.95
	Leaf petioles	26.30 ± 0.35	29.55 ± 0.20	25.27 ± 0.42	20.20 ± 5.66	25.3 ± 1.02	24.90 ± 0.75	25.93 ± 1.73	20.85 ± 1.07	23.00 ± 1.63
	Stolons	17.73 ± 1.28	22.60 ± 1.38	22.90 ± 1.55	18.43 ± 0.80	18.3 ± 0.35	16.43 ± 0.81	21.63 ± 0.84	15.73 ± 0.50	18.23 ± 2.17
	Roots	6.50 ± 0.75	8.47 ± 0.34	10.03 ± 0.77	5.85 ± 1.13	6.90 ± 0.30	8.03 ± 0.74	8.47 ± 0.69	5.97 ± 0.19	9.30 ± 0.51
Ca (g kg^{-1} DM)	Leaf blades	30.78 ± 1.38	24.73 ± 0.75	28.50 ± 3.15	28.53 ± 1.91	28.70 ± 1.28	25.83 ± 0.73	24.47 ± 0.42	28.53 ± 1.39	24.77 ± 2.22
	Leaf petioles	30.75 ± 0.84	31.05 ± 1.65	24.10 ± 0.44	25.90 ± 2.42	26.17 ± 0.32	29.80 ± 1.12	26.43 ± 0.32	24.80 ± 1.50	26.73 ± 2.35
	Stolons	4.90 ± 0.44	5.60 ± 0.20	10.17 ± 1.94	6.53 ± 0.41	5.30 ± 0.31	3.77 ± 0.09	5.10 ± 0.60	5.60 ± 0.21	11.57 ± 0.78
	Roots	5.55 ± 0.20	5.43 ± 0.27	7.37 ± 0.22	5.40 ± 0.29	4.50 ± 0.10	4.90 ± 0.47	5.20 ± 0.29	5.87 ± 0.58	4.57 ± 0.32
Mg (g kg^{-1} DM)	Leaf blades	4.30 ± 0.20	4.63 ± 0.27	5.60 ± 0.40	4.30 ± 0.20	3.70 ± 0.27	3.70 ± 0.21	3.43 ± 0.03	4.63 ± 0.15	3.67 ± 0.50
	Leaf petioles	3.90 ± 0.46	3.65 ± 0.14	3.77 ± 0.58	3.00 ± 0.30	2.33 ±0.27	3.03 ± 0.12	3.93 ± 0.44	5.10 ± 0.12	1.80 ± 0.21
	Stolons	1.77 ± 0.32	1.80 ± 0.25	3.40 ± 0.61	1.73 ± 0.35	1.10 ± 0.17	1.63 ± 0.12	1.63 ± 0.03	1.60 ± 0.10	2.17 ± 0.18
	Roots	5.10 ± 0.06	5.57 ± 0.33	10.77 ± 0.29	4.95 ± 0.38	3.70 ± 0.15	5.37 ± 0.52	5.10 ± 0.42	4.23 ± 0.17	3.23 ± 0.39
Zn (mg kg^{-1} DM)	Leaf blades	63.9 ± 1.3	104.8 ± 2.8	145.9 ± 9.0	75.1 ± 4.7	55.7 ± 1.8	70.9 ± 5.2	67.9 ± 5.3	74.6 ± 10.1	40.7 ±5.0
	Leaf petioles	22.7 ± 0.4	46.9 ± 0.1	30.7 ± 1.7	42.3 ± 12.7	23.9 ± 1.2	24.2 ± 0.8	29.8 ± 1.5	25.6 ± 0.7	22.7 ± 0.8
	Stolons	19.3 ± 0.6	50.7 ± 3.1	23.6 ± 0.2	30.2 ± 2.2	17.5 ± 0.7	21.1 ± 1.8	22.0 ± 0.9	25.3 ± 1.5	19.9 ± 1.4
	Roots	47.0 ± 0.7	66.5 ± 1.7	51.9 ± 4.6	55.4 ±2.2	40.1 ± 2.0	47.5 ± 5.1	48.7 ± 3.6	46.8 ± 2.4	37.3 ± 7.5
Fe (mg kg^{-1} DM)	Leaf blades	88.3 ± 9.3	119.8 ± 3.8	105.9 ± 7.8	69.3 ± 11.1	52.4 ± 3.1	53.0 ± 3.6	63.5 ± 9.7	51.3 ± 1.7	58.0 ± 9.5
	Leaf petioles	49.7 ± 2.1	58.8 ± 0.3	62.2 ± 2.5	55.6 ± 8.3	31.9 ± 1.3	51.6 ± 14.6	46.5 ± 0.5	35.8 ± 0.6	36.2 ± 13.3
	Stolons	38.8 ± 3.9	61.2 ± 4.8	49.2 ± 4.7	55.8 ± 6.4	23.1 ± 1.1	47.5 ± 5.1	43.3 ± 1.2	42.0 ± 2.7	32.1 ± 1.3
	Roots	94.6 ± 0.2	149.8 ± 6.0	118.4 ± 2.5	137.5 ± 15.5	47.6 ± 5.8	89.6 ± 5.0	86.8 ± 9.2	78.6 ± 12.0	63.3 ± 5.1
Cu (mg kg^{-1} DM)	Leaf blades	9.1 ± 0.4	7.5 ± 0.4	8.4 ± 0.4	6.3 ± 0.6	6.2 ± 0.2	10.2 ± 1.1	7.2 ± 0.5	8.3 ± 0.3	8.7 ± 0.6
	Leaf petioles	7.4 ± 0.1	7.2 ± 0.2	8.0 ± 0.4	5.3 ± 0.3	6.5 ± 0.2	10.5 ± 0.8	6.0 ± 0.2	8.4 ± 0.3	6.4 ± 0.5
	Stolons	9.0 ± 0.4	9.8 ± 2.0	9.2 ± 0.2	8.5 ± 1.0	6.2 ± 0.2	12.4 ± 0.3	6.8 ± 0.3	6.8 ± 0.5	7.1 ± 0.5
	Roots	24.1 ± 1.2	79.6 ± 14.4	122.1 ± 32.2	84.1 ± 15.6	56.0 ± 0.9	97.1 ± 27.5	111.9 ± 5.1	94.3 ± 1.9	56.5 ± 10.9
Mn (mg kg^{-1} DM)	Leaf blades	71.8 ± 5.2	168.1 ± 9.9	321.6 ± 29.9	238.6 ± 9.8	131.9 ± 9.2	162.5 ± 9.9	187.8 ± 24.4	168.5 ± 15.3	230.1 ± 45.8
	Leaf petioles	208.8 ± 13.4	47.4 ± 3.3	63.8 ± 8.1	57.4 ± 2.7	45.9 ± 6.6	53.5 ± 1.1	57.5 ± 4.6	58.0 ± 0.5	54.1 ± 7.5
	Stolons	55.9 ± 0.4	18.9 ± 2.8	26.2 ± 1.7	29.7 ± 2.5	18.1 ± 1.7	18.4 ± 2.0	19.3 ± 2.1	24.3 ± 1.2	21.5 ± 2.8
	Roots	58.6 ± 4.4	48.9 ± 4.7	58.3 ± 2.5	46.8 ± 5.5	42.1 ± 2.8	41.3 ± 5.9	45.7 ± 4.8	56.4 ± 2.7	45.0 ± 9.0

Values are means ± SE from three independently analyzed samples. DM, dry mass.
